# Author Index for Volume 102

**DOI:** 10.1038/sj.bjc.6605728

**Published:** 2010-06-08

Aaltonen, LA 447, 455

Abbotts, R 704

Abbruzzese, JL 144

Abdul-Ghani, R 361

Aboagye, EO 1555

Abrahamowicz, M 1113

Abramovitz, M 570

Abu-Khalaf, M 134

Aburatani, H 206

Adansa, JC 1687

Adenis, A 59, 1207

Aggarwal, S 1265

Agustsson, T 1541

Aitini, E 68

Aitken, JF 620

Akslen, LA 369

Al Olama, AA 414

Ala-Opas, M 469

Al-Attar, A 704, 1600

Albanell, J 1137

Albert, US 645

Alemao, E 1456

Alessi, S 1778

Ali, SH 1657

Alisa, A 748

Allerstorfer, S 1145

Al-Saffar, NMS 1

Altermatt, HJ 151

Altman, DG 173

Ambroisine, L 678

Ameri, K 561

Ammadi, RE 1627

Anastassiou, J 748

Anderson, N 1503

Andersson, C 383

Andre, F 462

Andreotti, G 1185

Anichini, A 1707

Annunziata, CM 495

Anson, K 249

Antoine, G 1284

Antoun, S 966

Arbyn, M 957

Ardizzoni, A 162

Arner, P 1541

Arnes, JB 369

Arriagada, R 213

Arslan, A 262

Arumi, M 1137

Arvanity, H 1762

Asakuma, J 1592

Asano, T 1592

Ashfield, A 719

Ashley, S 255, 995

Ateenyi-Agaba, C 262

Atkins, J 1311

Attard, G 678

Atula, T 892

Au, JSK 1731

Aultman, R 19

Auvinen, A 469

Avan, BI 1657

Avery, KNL 1335

Avila, H 774

Avril-Sassen, S 35

Azad, N 495

Azodi, M 134

Azzato, EM 1294

Azzouz, F 294

Baade, PD 620

Bache, M 731

Bachem, M 188

Bachmann, P 966

Backen, A 1524

Baek, S-Y 710

Bagga, S 1428

Bagherzadeh, A 541

Bah, M 1037

Bairaktari, E 1762

Baker, KL 1371

Baker, L 719

Balkissoon, J 1355

Balkwill, F 1555

Ballot, J 1157

Balmain, A 1555

Balzi, M 685

Bamberg, M 1213

Bando, Y 908

Bandres, E 987

Baneshi, MR 1503

Banks, MR 1608

Bao, S 789

Bao, Y 1422

Barbotte, E 181

Bartlett, JMS 1503

Barton, CA 87

Barwick, BG 570

Beauchamp, M-E 1113

Beesley, AH 1200

Begent, RH 1106

Behboudi, S 748

Behnam-Motlagh, P 383

Bell, J 1106

Bell, R 1099

Bellmunt, J 1137

Bellone, M 134

Bellone, S 134

Bellosillo, B 1137

Beloueche-Babari, M 1

Bénard, J 1032

Bengala, C 68

Bennouna, J 59

Benoy, IH 276

Ben-Shlomo, Y 249

Benson, VS 1654

Bentley, J 342

Benvenuti, S 685

Beral, V 1654

Berchery, D 966

Berg, CD 220

Berger, W 1145

Berle, EJ 957

Berlin, JA 301

Berney, DM 678

Berrington de Gonzalez, A 220

Berry, DA 651

Berthier, A 1024

Bertolini, F 68

Bertorelle, R 1300

Best, LA 1180

Bevan, R 1428

Bhatti, R 995

Bhoopathi, P 530

Bian, X-w 1052

Bianco, R 513

Bidart, JM 462

Bidstrup, PE 1348

Bihl, MP 151

Bijarchi, R 237

Billay, EM 957

Binta-Kahwa, J 262

Birse, CE 1265

Birse-Stewart-Bell, L-J 719

Bishop, H 285

Bitarte, N 987

Black, F 553

Blay, J-Y 1032

Blazeby, JM 1335

Bobin, JY 1024

Boddy, AV 1003

Boeck, S 1308

Boffetta, P 1190

Bogdanovic, G 796

Bolaños, M 1468

Bolognesi, M 1707

Bonasoni, MP 162

Boni, C 68

Bonnetaud, C 1627

Bonodeau, F 1207

Bonvalot, S 1032

Boormans, JL 1491

Borel Rinkes, IHM 1254

Borge, B 957

Borsboom, GJJM 27

Bosch, C 1468

Boswell, S 748

Bouley, DM 561

Boulle, N 181

Bounds, R 693

Bourdon, J-C 719

Bouzyk, M 570

Boven, E 803

Bowers, P 301

Bown, SG 1608

Boxall, J 1355

Boyd, DD 774

Boyer, JC 1080

Boyer-Guittaut, M 1024

Bozzetti, C 162

Bradley, MC 1415

Brahmer, J 1311

Brandner, S 784

Brandt, A 42

Bray, J 1003

Breit, SN 665

Brennan, P 1190

Brenner, H 447, 455, 1411

Brewer, D 678

Brewster, DH 930, 1190, 1661

Bridges, K 301

Bright, SA 1474

Brinkmann, K 1717

Brinton, LA 1185

Broderick, P 447, 455

Brodie, AH 815

Brodin, D 1541

Broecker- Preuss, M 376

Broggini, M 104

Brookes, K 1524

Brossart, P 1213

Brouste, V 1032

Brown, DA 665

Brown, G 255

Brown, MD 403

Brown, TP 1428

Broxterman, HJ 268

Bruder, G 1555

Buch, S 447, 455

Buckley, DL 23

Buclin, T 1198

Buffa, FM 428

Bulman, B 1091

Bunch, KJ 615

Burbos, N 1201

Burcia, V 181

Burger, AM 815

Burgess, R 1311

Burkinshaw, R 1099

Bushmakin, AG 658

Butt, E 1645

Buza, N 134

Byrne, M 73

Cadman, L 1405

Caldas, C 1294

Calvert, AH 553

Camburn, T 995

Cameron, D 1099

Cameron, K 1254

Camisa, R 162

Campbell, F 577

Campbell, H 447, 455

Campbell, NC 1447

Campiani, G 1474

Cañadas, I 1137

Candiotto, C 1300

Cantini, LP 1724

Cantwell, MM 1415

Cao, D 294

Capelletti, M 1495

Caplin, ME 1106

Cappelleri, JC 658

Caradec, J 1037

Cardon, LR 1371

Carey, FA 693

Cargnelutti, M 134

Carozza, SE 227

Carpenter, L 1085

Carracedo, A 447, 455

Carrington, BM 23

Carroll, P 1474

Cartier, C 181

Carvajal-Carmona, L 447, 455

Casagrande, F 134

Cassell, JA 947

Castañón, A 933

Castells, A 447, 455

Castellví-Bel, S 447, 455

Castex, F 852

Castleberry, AW 627

Castro-Carpeño, J 1468

Cavalli, F 673

Cavenagh, J 1795

Cazier, JB 1044

Cazin, J-L 1207

Ceelen, WP 837

Cella, D 658

Cellai, I 685

Cempaka, NL 1511

Ceni, E 685

Cerny, T 673

Chan, AT 1079

Chan, K-K 332

Chan, K-W 332

Chan, S 704, 995

Chan, WY 419

Chan, Y-P 332

Chao, M 1511

Chao, R 1311

Chao, T-Y 981

Chaplin, DJ 1355, 1555

Chaplin, T 1044

Charbonneau, C 658

Chatterjee, M 1578

Chau, I 255

Chelle, F 966

Chen, EP 1371

Chen, G 188, 1753

Chen, J 351, 1052, 1185

Chen, K 1052

Chen, M 1371

Chen, P-J 981

Chen, Q 940

Chen, Y-Y 594

Cheng, A-L 981

Cheng, L 541

Cheng, SQ 1618

Cheng, WW 1731

Cherrie, JW 1428

Chetty, C 530

Cheung, HH 419

Cheung, P 1391

Chia, K-S 1190

Chimingqi, M 1037

Chinegwundoh, F 249

Chiozzotto, D 104

Chirimwami, RB 957

Chiu, Y-T 332

Chiyomaru, T 883

Cho, E 489

Cho, WCS 1731

Chopitea, A 987

Chow, EJ 227

Chow, T-FF 1244

Chow, W-H 1676

Chua, C-W 332

Chuang, S-C 1190

Chumsri, S 815

Chung, Y-L 1

Cibas, ES 1495

Clark, J 678

Clark, SJ 87

Clarke, N 403

Clay, TM 124

Clement, K 1541

Clements, K 285

Clifford, GM 1657

Clisant, S 1207

Clynes, M 1157

Coates, AS 1341

Cocco, E 134

Cocker, H 1461

Cohn, SL 1319

Coindre, J-M 1032

Cole, M 1003

Coleman, MP 1438

Coleman, RE 1010, 1099

Collin, F 1032

Collins, D 1157

Collins, K 1461

Colt, JS 1676

Conneally, E 1474

Conroy, T 59

Conte, P 68

Conti, M 1037

Conway, C 1157

Cooke, TG 1503

Cooper, CS 678

Corbishley, C 249

Costelloe, CM 651

Costes, V 181

Coutelle, O 1717

Cox, B 1665

Cozzolino, R 513

Crack, L 727

Crafa, P 162

Crampette, L 181

Crawford, J 301

Cremolini, C 1076

Crown, J 1157

Cruz, JJ 1687

Csajka, C 1198

Cubie, HA 930, 1405

Cummings, J 1524

Cummins, MM 475

Cunningham, D 255

Curtin, NJ 342

Curtis, RE 220

Cuschieri, K 930

Cuzick, J 678, 933, 1405

Daayana, S 1129

Dahl, E 1736

Dahlman, I 1541

Dahlman-Wright, K 1541

Dai, Y 1549

Dalenc, F 1081

D’Alessio, G 513

Dalgleish, AG 115

Dalmases, A 1137

Dalton, SO 1348

Damiano, V 513

Dansin, E 1207

Datchary, J 1024

Dattani, M 1519

Davidovits, A 1361

Davidson, N 995

Davidson, SE 23

Davis, AJ 419

Davis, FG 1676

Dawson, J 1091

De, P 1553

de Bekker-Grob, EW 972

de Bono, JS 678

de Bruijn, MT 1254

de Haan, MC 1400

de Haas, RR 268

de Koning, HJ 27

De Laroche, G 1024

de las Morenas, A 1284

De Lorenzo, C 513

De Rossi, A 1300

de Souza, P 1456

de Verbizier, D 181

Dealis, C 68

Dean, EJ 97

Deans, DAC 665

Debergh, I 837

Decker, T 500

Decosterd, LA 1198

Dégardin, M 1207

Deguchi, K 1174, 1378

Deininger, MW 1474

Del Bianco, P 1300

Del Giovane, C 68

Delage-Mourroux, R 1024

Delaloge, S 213, 462

Delattre, O 1769

Deledda, C 685

Delia, D 1707

Deltour, I 1348

den Hollander, M 1699

Depenni, R 68

der Maase, H 867

Desai, M 1405

Desauw, C 1207

Descotes, F 1024

Desné, S 966

Devi, G 124

Devilee, P 447, 455

Dewar, JA 719

Dey, N 1553

Diamandis, M 1244

Diebold, J 151

Dietel, M 1736

Dinh, DH 530

Dirix, LY 276

Dive, C 8, 97, 577, 1524

Djuzenova, CS 1578

Dobrovic, A 1219

Dodwell, D 1099

Doherty, VR 1661

Doi, Y 898

Doki, Y 1483

Dolly, S 629

Dômont, J 1032

Donaldson, SB 23

Donkers, B 972

Donovan, JL 414, 1335

Donskov, F 867

dos Santos Silva, I 1438

Dose-Schwarz, J 35

Double, JA 1555

Douillard, JY 59

Down, L 1335

Downes, CS 1080

Dowsett, M 1235

Drago, C 1707

Ducreux, M 59

Dufresne, A 1032

Dunbar, CE 1636

Dunlop, M 447, 455

Dunlop, NM 1052

Dunn, JM 1608

Dunne, P 1080

Eagleton, N 1753

Easton, D 414

Eccles, SA 1555

Echeverry, MM 447, 455

Eckert, AW 731

Edwards, J 1503

Edwards, S 678

Eeles, R 414

Egorov, VI 1533

Eguchi, H 1483

Eigentler, T 1213

Elkord, E 1129

Ellens, HE 1371

Ellis, I 285

El-Sahwi, K 134

Elst, HJ 276

El-Tanani, M 332

Emptas, H 1074

Endlicher, E 500

Engvall, M 796

Enokida, H 883

Epstein, RJ 981, 1391

Erlandsson, F 827

Escaff, S 922

Escudero, P 1468

Escudier, B 80

Espinosa, I 561

Essink-Bot, ML 27, 972

Eustace, AJ 1157

Evans, A 285

Evans, AH 1235

Evans, DB 1235

Evans, DGR 1091

Evans, S 249

Everitt, J 1555

Fahey, T 48

Falasca, M 104

Falck-Miniotis, M 1

Falcone, A 1076

Falewee, MN 966

Falk, GA 48

Fang, DD 1265

Faragalla, H 1244

Faraoni, P 685

Farber, RA 1080

Fareed, KR 704, 1600

Faria, A 629

Farmer, I 1235

Farningham, DAH 1555

Faroux, R 59

Fear, NT 615

Fearon, KCH 665

Fedorenko, IV 1724

Feliu, J 1468

Fend, F 500

Feng, YX 1618

Fernández, JM 922

Fernando, N 727

Ferreri, AJM 673

Ferrero, J-M 1081

Field, H 414

Fiorentino, M 1495

Firth, MJ 1200

Fischer, H 1145

Fisher, G 678

Fitzpatrick, P 456

Flaherty, KT 1724

Flentje, M 1578

Fletcher, A 678

Flockhart, DA 294

Flohr, P 678

Flores, LM 1495

Folprecht, G 500

Fontana, A 68

Ford, J 1200

Forestier, E 796

Försti, A 447, 455

Fortunato, L 1428

Foster, CS 678

Foster, NR 165

Fournier, C 1207

Fowler, DW 115

Fox, EE 227

Fragoulis, EG 1384

Fraichard, A 1024

Franceschi, S 262, 1312, 1657

Francken, AB 1445

Frattini, M 151

Fraumeni Jr, JF 1185

Frederiksen, K 1348

Freitas, JR 1200

Frick, H 151

Frietsch, JJ 1645

Friis, S 1190

Fritzer-Szekeres, M 1361

Fritzsche, FR 1736

Fuchs, CS 1079, 1422

Fujii, H 520

Fujimura, L 883

Fujiwara, H 1174, 1378

Fukuhara, H 1061

Furlong, F 456

Furukawa, Y 325

Füssel, S 731

Fuyuhiro, Y 898

Gabril, M 1244

Gacía-Foncillas, J 987

Gaggioli, C 392

Gagnon, B 1113

Galamb, O 765

Gallant, G 506

Gallen, M 1137

Galli, A 685

Gallinger, S 447, 455

Ganesan, TS 1355

Ganju, V 475

Gao, Y 1052

Gao, Y-T 1185

Garbe, C 1213

García-Girón, C 1468

Gardner, P 403

Garfield, DH 1309

Garner, E 577

Garrel, R 181

Gauglhofer, C 1145

Gavish, M 1180

Gaya, A 1355

Gazi, E 403

Gebski, V 475, 1341

Geisler, C 1736

Gekiere, JP 966

Gelardi, T 513

Gelderblom, H 1699, 1791

Geleff, S 1361

Gelmini, S 685

Genève, J 1081

Genre, D 1081

Georgoulias, V 1762

Gerald, WL 678

Gerhardt, J 1736

Germer, M 151

Ghaneh, P 577

Giammarile, F 1319

Giamougiannis, P 1201

Giarenis, I 1201

Gibbon, K 1044

Gibbs, D 475

Giessrigl, B 1361

Gil, M 1099, 1468

Gil-Arnaiz, I 1687

Gilbert, E 220

Gillatt, D 249

Gillbanks, A 255

Gillmore, R 1106

Giovannucci, E 1422

Giovannucci, EL 1079

Girgis, A 1244

Gislefoss, RE 1665

Glaspy, J 301

Glenn, WK 788, 1551

Glennie, MJ 1555

Gloss, BS 87

Glynne-Jones, R 1123

Godage, HY 104

Golakoti, T 1361

Gong, W 1052

González, JM 922

González, LO 922

González-Barón, M 1468

González-Reyes, S 922

Gooding, LR 796

Gore, M 80

Gorman, D 1661

Gorman, S 456

Gorr, TA 1736

Gossage, L 704

Gotoh, N 206

Govindan, R 1311

Grabellus, F 376

Grabsch, H 1519

Grabsch, J 1519

Graham, C 930

Graham, K 1284

Graham, P 1665

Grannis Jr, FW 627

Granström, C 1786

Grasl-Kraupp, B 1145

Graubard, BI 1676

Graziano, F 1076

Green, AC 620

Green, C 234

Green, J 1654

Green, T 1461

Greenhalf, W 577

Greither, T 731

Grell, K 1670

Greystoke, A 1524

Gridling, M 1361

Griffin, JD 351

Griffin, MJ 1003

Grillo, F 1106

Grimm, C 952

Grunewald, TGP 1645

Grusch, M 1145, 1361

Guchelaar, H-J 1791

Guerrier, B 181

Guerrieri, R 1224

Guillou, L 1032

Gujrati, M 530

Gullo, G 1157

Guo, WX 1618

Guruge, D 727

Gustafson, G 1670

Gustafsson, B 796

Guy, M 414

Györffy, B 361

Haanen, JBAG 803

Habbema, JDF 972

Hacker, NF 87

Hackshaw, A 1106

Hadji, P 645

Haglund, C 892

Hagström, J 892

Hall, A 414

Hall, M 1724

Hamaoka, T 651

Hamaya, Y 916

Hamberg, P 1699

Hamburger, AW 815

Hamdy, FC 414, 1335

Hamilton, AL 1219

Hammond, S 124

Hampe, J 447, 455

Hampton, JM 799

Han, M-E 710

Han, T-Q 1185

Hanby, A 285

Handolias, D 1219

Hankinson, SE 489

Hannaford, PC 1447

Haouala, A 1198

Hardie, A 930

Harris, AL 428, 561

Harrison, M 456

Hart, C 403

Hartley, R 1091

Harvey, R 810

Harwood, CA 1044

Hashibe, M 1190

Hassall, T 620

Haubert, D 1717

Havet, K 1627

Hayakawa, M 1592

Hayes, DF 294

Häyry, V 892

He, T 1265

Hebbar, M 59

Heddleston, JM 789

Hefler, L 952

Hefler-Frischmuth, K 952

Hegewisch-Becker, S 500

Heinemann, V 506, 1308

Heinimann, K 151, 1068

Heinzle, C 1145

Hemminki, K 42, 447, 455, 1190, 1397, 1786

Heng, B 788, 1551

Henne-Bruns, D 188

Hennekam, FAM 1400

Henry, D 301

Henry, NL 294

Herbacek, I 1361

Hercbergs, A 1309

Herterich, S 1645

Hinohara, K 206

Hiraike, H 1061

Hirakawa, K 844, 898

Hishida, A 916

Hitt, R 1687

Hjelmeland, AB 789

Ho, JWC 447, 455

Ho, L 1405

Hobeika, A 124

Hochhauser, D 1106

Hoekstra, HJ 1445

Hoekstra-Weebers, JW 1445

Hofman, P 1627

Hofman, V 1627

Hofsli, E 482

Hofstra, RMW 447, 455

Hol, L 972

Holen, I 1010

Hollis, BW 1079

Holmes, MD 489

Holmes, P 1428

Holzhausen, H-J 731

Holzmann, K 1145

Honig, A 1645

Honkaniemi, E 796

Hoogwater, FJH 1254

Hooper, S 392

Horcic, M 151

Horel, S 227

Horiguchi, A 1592

Hortobagyi, GN 651

Hosono, T 873

Hou, L 1185

Houghten, R 97

Houlston, RS 447, 455

Houston, N 495

Hovland, S 957

Howard, OMZ 1052

Howell, A 1091

Høybye, MT 1348

Hoyle, M 234

Hsiao, L-T 981

Hsing, AW 1185

Hsu, C 981

Hsu, C-H 981

Hsu, D 124

Hu, B 862

Hu, HS 1618

Hu, L 1276

Huang, J 1052

Huang, L 1371

Huang, W 796

Hudson, T 447, 455

Huget, P 276

Hughes, CM 1415

Hughes, TA 1746

Hui, P 134

Huitema, ADR 673

Hulman, G 1405

Hunter, M 665

Hur, G-Y 710

Husslein, H 952

Hutchings, SJ 1428

Hutchins, G 1519

Huttary, N 1361

Ibrahim, F 249

Ibrahim, N 827

Ichikawa, D 1174, 1378

Iglesias, M 1137

Ikuma, M 916

İlie, M 1627

Imanaka, N 351

Imrie, J 930

Irigoyen, A 1687

Isagawa, T 206

Isaksson, B 1541

Ishiguro, T 774

Isla, D 1687

Islam, R 651

Ito, K 1592

Jaffe, RB 1276

Jäger, W 1361

James, R 1123

Jamieson, D 1003

Jänicke, F 35

Jänne, PA 1495

Jasper, S 1645

Jayne, DG 1746

Jayson, GC 8, 1524

Jeffrey, SS 561

Jellum, E 1665

Jensen, HK 867

Jensen, MR 1578

Jensen, S 1661

Jeon, T-Y 710

Jeppesen, AN 867

Ji, J 1397

Jia, H 541

Jitlal, M 1123

Joerger, M 673

Johansen, C 1348, 1670

Johansson, A 383

Johansson, D 383

Johnson, N 342

Johnston, SRD 995, 1235

Jonasson, JG 1190

Jones, AP 23

Jones, L 727

Jordan, LB 719

Jost, F 19

Jouvenot, M 1024

Jovine, E 1306

Jukkola-Vuorinen, A 1018

Julian-Reynier, C 1081

Kadima, TM 957

Kalder, M 645

Kameda, C 738

Kamitani, T 873

Kammerer, U 1645

Kamper-Jørgensen, M 1670

Kanaoka, S 916

Kang, Y 457

Kansikas, M 1068

Kantelinen, J 1068

Kanzler, S 506

Kapp, M 1645

Kappler, M 731

Karami, S 1676

Karanjia, N 255

Karihtala, P 1018

Kariola, R 1068

Karlsen, F 957

Karner, J 1145

Kashiwagi, S 898

Kashkar, H 1707, 1717

Kasymjanova, G 1113

Katano, M 738

Kato, S 1061

Kato, Y 898

Kauppila, S 1018

Kavanah, M 1284

Kawaguchi, Y 520

Kawahara, K 883

Kawajiri, H 844

Kayani, I 1106

Kaye, PV 1600

Ke, S 1753

Keane, MM 754, 1099

Kearins, O 285

Kees, UR 1200

Kelland, LR 1555

Keller, G 500

Kennedy, S 1157

Keogan, M 73

Kerbrat, P 1081

Kerjaschki, D 1361

Kerr, L 1219

Keski-Säntti, H 892

Keumeugni, C 1037

Khakpour, N 815

Khan, NF 1085

Kikkawa, N 883

Kilpeläinen, TP 469

Kim, B-S 710

Kim, C 1327

Kim, D-Y 1692

Kim, G-H 710

Kim, H-J 710

Kim, J 436

Kim, J-B 710

Kim, J-H 1692

Kim, J-Y 710

Kim, S 436

Kim, ST 658

Kim, YJ 1265

Kim, Y-M 1692

Kim, Y-T 1692

Kindelberger, DW 1495

King, C 1284

King, JC 615

Kirby, R 249

Kirkwood, A 1106

Kitchener, HC 1129, 1405

Kleter, B 262

Kliewer, EV 1190

Klimpfinger, M 1145

Klinger, R 456

Knox, F 1091

Knüchel, R 1736

Kobayashi, S 206, 1483

Kockler, L 59

Kodani, M 570

Koessler, T 447, 455

Kogner, P 1670

Koh, W-P 610

Köhler, S 602

Kohn, EC 495

Köhne, C-H 506

Komatsu, S 1174, 1378

Konishi, H 1174, 1378

Kono, K 520

Kontos, CK 1384

Kopantseva, MR 1533

Kopantzev, EP 1533

Kopetz, S 144

Korhonen, MK 1068

Kornmann, M 188

Korsten, H 1491

Kosuga, T 1174, 1378

Kote-Jarai, Z 414

Kotz, H 495

Kotzsch, M 731

Koumakpayi, IH 1163

Kovac, M 151

Kovacs, G 678

Koyama, S 1061

Krähenbühl, S 673

Krajewska, E 594

Kranenburg, O 1254

Kreisman, H 1113

Kremer, R 1180

Krenács, T 765

Krishna, S 196

Kristiansen, G 1736

Krönke, M 1717

Krop, IE 1495

Krupitza, G 1361

Kubo, M 738

Kudo, E 908

Kuipers, EJ 972

Kujala, P 469

Kulyte, A 1541

Kuroda, K 1592

Kuroda, M 206

Kyembwa, L 957

Laccetti, P 513

Lacombe, J 181

Lacroix, L 462, 1032

Ladenstein, R 1319

Ladhoff, A-M 1736

Laffins, B 1753

Lagrasta, CA 162

Lähteenmäki, P 1670

Lakka, SS 530

Lalloo, F 1091

Lambea, J 1687

Lamy, P-J 1074

Lane, JA 1335

Langendijk, JA 1778

Larkin, AM 1157

Larochelle, A 1636

Lasithiotakis, K 1213

Lathia, JD 789

Lau, S 1391

Lau, ST 583

Laudico, A 1411

Lautenschläger, C 731

Lawler, M 1474

Lawrence, G 285

Lawson, JS 788, 1551

Le, M 165

Lê, MG 213

Le Cesne, A 1032

Le Page, C 1163

Leach, MO 1

Lebeau, A 35

Lecis, D 1707

Lecumberri, MJ 1687

Ledermann, J 1123

Lee, AJ 1447

Lee, AJX 1294

Lee, CK 1341

Lee, CN 1265

Lee, H-P 610

Lee, HY 941

Lee, JHK 1551

Lee, J-M 495

Lee, TL 419

Lee, Y-CA 1190

Lee, Y-S 710

Lee, Y-W 710

Leese, MP 316

Leigh, IM 1044

Leipold, H 952

Leiter, U 1213

Leivo, I 892

Lema, L 1137

Leongamornlert, D 414

Lerch, MM 447, 455

Leroux, A 1032

Leung, PS 583

Lewington, V 1319

Lewis, CE 594

Leyland-Jones, B 570, 815, 1553

Leyvraz, S 1198

Li, JZ 658

Li, L 294

Li, N 1618

Li, X 1397

Li, Z 789

Liao, R-Y 940

Lie, AK 957

Ligon, AH 1495

Lim, B 436

Lin, S-Y 774

Lin, ZX 583

Lin, Z-Z 981

Lind, JSW 268

Lindblom, A 447, 455

Linder, K 1541

Lipton, L 447, 455

Liu, P 651

Liu, SR 1618

Liu, WM 115

Liu, X 1549

Liu, Z 188, 234

Llédo, G 59

Llombart, A 1468

Lobo, DN 704, 1600

Lockyer, N 403

Loda, M 1495

Loos, WJ 1699

López, R 1468

Lopez-Crapez, E 1074

Lord, SJ 1341

Lordick, F 500

Lorenzen, S 500

Lorenzo Bermejo, J 42

Loric, S 1037

Losa, F 1468

Loughery, J 1080

Loupakis, F 1076

Lovat, LB 1608

Lu, X 457

Luber, B 500

Luciani, P 685

Ludwig, H 301

Luesley, D 1405

Lugli, A 151

Lüke, C 1736

Lundin, J 892

Lunec, J 553

Luong, R 561

Luppi, G 68

Lyerly, HK 124

Määttänen, L 469

Macartney, J 285

MacCarthy, A 615

MacDonald, N 1113

Macefield, RC 1335

Machavoine, C 462

Mackenzie, GD 1608

Madan, J 1461

Madewell, JE 651

Madhok, BM 1746

Madhusudan, S 704, 1600

Madlener, S 1361

Madroñal, C 1468

Maes, H 276

Maffucci, T 104

Magdolen, V 731

Maget, B 966

Mahalingam, D 754

Maher, SG 73

Mahgoub, T 1157

Mahner, S 35

Maia, AT 1294

Makeieff, M 181

Makin, G 97

Mäkinen, LK 892

Mäkitie, A 892

Malavasi, N 68

Malavige, GN 727

Malik, FR 1657

Mallett, S 173

Mancini, J 1081

Mange, A 181

Mangtani, P 1438

Manivet, P 1037

Mankaruous, M 1244

Manzoni, L 1707

Mao, C 940

Marcus, R 19

Marcussen, N 867

Marian, B 1145

Maringe, C 1438

Marshall, SJ 1099

Martin, A-L 1081

Martin, L-A 1235

Martinez-Aviles, L 1137

Martinez-Fernandez, A 1137

Martinez-Trufero, J 1687

Martos, C 1190

Marubashi, S 1483

Maruyama, T 520

Masetti, M 1306

Mastik, MF 1778

Mastrangelo, E 1707

Matar, C 1037

Matsuda, K 447, 455

Matsumoto, Y 1061

Matsuoka, J 898

Matthay, KK 1319

Maudelonde, T 181

Mavroudis, D 1762

Mayer, A 1106

Maylevin, F 1081

Mazure, NM 1627

Mazzoletti, M 104

Mazzucchelli, L 151

McArthur, C 1276

McArthur, GA 1219

McCann, A 456

McCowan, C 48, 719

McCrudden, CM 332

McDaid, JR 1080

McElligott, AM 1474

McGrath, S 810

McKay, MJ 1511

McKinnon, K 1265

McLaughlin, CC 227

McShane, P 1519

Meadows, H 1123

Medema, JP 1254

Medina, VM 1411

Meier, F 1213

Meijer, CJLM 957, 1657

Meijerink, MR 803

Mejhert, N 1541

Mejia-Guerrero, S 1244

Mendez, J 1284

Menon, U 1405

Mesher, D 1405

Meslier, M 966

Mes-Masson, A-M 1163

Mesmer, D 1265

Messina, JL 1724

Metcalfe, C 249, 1335

Meuric, J 966

Meye, A 731

Meyer, T 1106

Meyerhardt, JA 1079

Michaelson, MD 658

Michaud, DS 1422

Michels, KB 1400

Michon, J 1319

Mickisch, G 80, 232, 1078

Mihály, Z 361

Mikulits, W 1361

Miles, D 995

Millan, D 930

Miller, CJ 428

Miller, M 1284

Mills, GB 941

Mimura, K 520

Minasian, L 495

Mineur, L 1074

Mir, D 827

Mirasol-Lumague, MR 1411

Missiaglia, E 1769

Miwa, A 844

Miyamoto, Y 1061

Mizukami, Y 520

Moch, H 1736

Moehler, M 506

Mograbi, B 1627

Mohammed, M 704

Moharer, J 383

Molinari, F 151

Møller, H 678

Molnár, B 765

Montagut, C 1137

Montani, M 151

Montemurro, M 1198

Montgomery, KD 561

Moodie, K 1219

Moore, PA 1265

Mooser, VE 1371

Moragon, E 1137

Moran, A 1091

Moreno, CS 570

Moreno, V 447, 455

Mori, M 1483

Morimura, R 1174, 1378

Morin, M 852

Morreau, H 447, 455

Morris, EP 1201

Morrison, J 414

Morse, MA 124

Mortier, L 1207

Mosse, CA 1608

Motzer, RJ 658

Mouroux, J 1627

Moutereau, S 1037

Moxham, T 234

Mudan, SS 255

Mueller, BA 227

Mueller, W 1519

Muir, G 249

Muir, K 414

Mukherjee, G 196

Mukwege, D 957

Munkácsy, G 361

Munko, AC 1724

Munuo, SS 1676

Murakami, M 1483

Murchie, P 1447

Murohashi, M 206

Murphy, MFG 615

Murphy, S 73

Murray, G 930

Murray, LJ 1415

Murtola, TJ 469

Murukesh, N 8

Musonda, P 1201

Mutch, DM 1541

Mvula, TM 957

Myerson, JS 629

Nagano, H 1483

Nagarajan, S 196

Nagasaka, K 1061

Nagler, RM 1180

Nakagawa, K 1061

Nakagawa, M 883

Nakagawa, S 1061

Nakamura, M 738

Nakamura, T 774

Nakamura, Y 325, 447, 455

Nakanishi, T 815

Nakasono, M 908

Nakatani, T 873

Nalwoga, H 369

Nam, J-H 1692

Nam, R 570

Napolitano, G 1415

Nargund, V 249

Narod, SA 237

Nathan, PD 1355

Natoni, A 754

Navaratnam, V 1555

Neal, DE 414, 1335

Nedergaard, J 1541

Negri, FV 162

Neoptolemos, JP 577

Neuville, A 1032

Neuzil, J 1224

Neville-Webbe, HL 1010

Newcomb, PA 799

Newell, DR 342

Newman, SP 316

Ng, K 1079, 1422

Nguyen, A 294

Nguyen, B 852

Nicholson, A 947

Nicoll, S 930

Nicolson, MC 1447

Nieto, JJ 1201

Niittymäkix, I 455, 447

Nishii, T 898

Nishiyama, K 883

Nitti, D 1300

Noda, S 898

Noda, T 1483

Norman, AR 255

Northover, J 1123

Norton, J 1265

Nouras, H 995

Novelli, MR 1608

Novello, S 1311

Nuijten, M 80, 232, 1078

Nutt, JE 553

Nyström, M 1068

Oberlin, O 1769

O’Brien, L 414

O’Brien, MER 629

O’Brien, PM 87

O’Connell, JW 1474

O’Connell, MJ 165

O’Connor, D 456

O’Connor, JP 23

O’Connor, L 1474

Oda, K 1061

O’Donnell, JS 73

O’Driscoll, L 1157

Ogg, GS 727

Oglesby, I 1157

Ognjanovic, S 227

Oh, S-O 710

Ohira, M 898

Okamoto, K 1174, 1378

Olde Bekkink, M 48

Ollila, S 1068

Olsen, JH 1786

Onishi, H 738

Ooi, WS 1157

Ornelles, DA 796

Ortholan, C 1627

Osada, T 124

O’Sullivan, J 456

Othieno, E 262

O’Toole, K 553

Otsuji, E 1174, 1378

Oukrif, D 1608

Ouyang, N 1753

Overman, MJ 144

Pallardy, S 1657

Pallares, C 1311

Pancholi, S 1235

Pander, J 1791

Pandite, L 1371

Panupinthu, N 941

Papadatos-Pastos, D 1762

Papadopoulos, IN 1384

Papadopoulou, A 1106

Paraiso, KHT 1724

Park, J-Y 1692

Parsons, SL 1600

Passos-Coelho, JL 1099

Pastorino, U 1681

Patel, B 249

Patel, J 1600

Patel, S 1769

Paterson-Brown, S 665

Pathan, AA 748

Patiño-Garcia, A 987

Patten, N 719

Pattje, WJ 1778

Pattyn, P 837

Pawlita, M 1129

Peach, G 1327

Pearson, ADJ 1319

Pecorelli, S 134

Pedrazzi, G 162

Peeters, D 276

Peeters, M 837

Peeters, PHM 1400

Pegram, M 827

Penegar, S 447, 455

Penel, N 1207

Péraldi-Roux, S 852

Peran, I 1549

Perera, KU 1200

Perera, M 727

Peri, A 685

Permert, J 1541

Perry, SL 1746

Persad, R 249

Pervez, S 1657

Peschel, C 500

Peterlongo, P 447, 455

Petrangolini, G 685

Petrovic, N 1541

Pharoah, PD 447, 455, 1294

Philips, S 294

Pidgeon, GP 73

Pierron, G 1769

Pikunov, M 1533

Pinder, SE 285

Pinilla, C 97

Pirie, K 1654

Pivot, X 827

Plummer, ER 553

Poiree, B 966

Polterauer, S 952

Pompe-Kirn, V 1190

Ponder, BAJ 1294

Pongratz, C 1717

Ponz-Sarvise, M 987

Popat, S 629

Popert, R 249

Popescu, R 1361

Porta, C 1196

Potter, BVL 104, 316

Poupard, L 1355

Pouysségur, J 1627

Powell, AA 561

Pozadzides, J 144

Pratesi, G 685

Prencipe, M 456

Pressoir, M 966

Previdi, S 104

Primrose, JN 1313

Pritchard-Jones, K 1769

Proby, CM 1044

Procopio, G 80

Prokop, M 27

Prové, A 276

Przyborek, M 506

Pucciarelli, S 1300

Puglisi, M 629

Puistola, U 1018

Pukkala, E 1190, 1786

Punt, CJA 1791

Purdie, CA 719

Purdie, KJ 1044

Purdue, MP 1676

Purkayastha, S 1327

Purohit, A 316

Purvis, J 1456

Puumala, S 227

Qian, ZR 908

Qu, W 87

Quinlan, PR 719

Quinn, AE 553

Quint, W 262

Quirke, P 1519

Raats, DAE 1254

Rabinovitch, PS 1608

Rachet, B 1438

Radice, P 447, 455

Rae, JM 294

Raja, EA 1447

Ramdass, B 196

Ramirez, N 987

Rampazzo, E 1300

Ranchère, D 1032

Ranson, M 97

Rao, JS 530

Rao, P 1769

Rao, S 301

Rashid, A 1185

Raza, SA 1657

Razak, ARA 553

Rebischung, C 59

Rebuffat, SA 852

Reck, BH 1371

Redaniel, MT 1411

Reed, MJ 316

Reed, MW 1461

Rego, RL 165

Rehman, M 196

Reid, AHM 678

Reinthaller, A 952

Renard, M 1769

Reni, M 673

Rennert, OM 419

Reuter, VE 678

Revaud, D 1037

Reynolds, JV 73

Reynolds, P 227

Rich, JN 789

Richardson, A 1665

Richter, U 602

Ricordati, C 685

Riegel, A 1549

Riley, AM 104

Ritchie, D 1099

Robarge, J 294

Robert, B 852

Roberts, T 285

Robinson, T 1461

Robinson, V 1555

Robson, CN 342

Roché, H 1081

Roden, R 1129

Rodrigues, I 966

Rodríguez, J 987

Rodriguez-Lafrasse, C 1024

Rofael, Y 1244

Rogers, G 234

Rohanachandra, LT 727

Rojo, F 1137

Rollag, H 1665

Ron, E 220

Roninson, IB 456

Roodenburg, JLN 1778

Rosa, R 513

Rose, M 1736

Rose, PW 1085

Rosell, R 1311

Rosenberg, CL 1284

Ross, DD 815

Ross, JA 665

Rossignol, G 966

Röthling, N 500

Rovira, A 1137

Rowe, PSN 862

Royer, B 827

Ruben, SM 1265

Rubino, C 213

Ruiz-Garcia, E 462

Ruiz-Ponte, C 447, 455

Rushton, L 1428

Russell, ST 833

Rustin, GJ 1355

Ruterbusch, JJ 1676

Rutherford, TJ 134

Ruzzo, A 1076

Ryd, W 957

Rydén, M 1541

Ryder, S 1555

Saad, F 1163

Saez, S 1024

Safont, MJ 1468

Sahai, E 392

Saiko, P 1361

Saji, S 1061

Sakai, M 325

Sakoda, LC 1185

Salas, S 1032

Salcido, CD 1636

Salemi, R 1219

Saletti, P 151

Salud, A 1468

Samali, A 754

Sandstrom, RE 786

Sangster, K 665

Sano, T 908

Santarelli, L 1224

Santin, AD 134

Sargent, DJ 165

Saridaki, Z 1762

Sariola, H 892

Sarker, D 1106

Sarosy, GA 495

Sasco, AJ 1024

Sasieni, P 933, 1405, 1608

Sato, A 1592

Sauerbrei, W 173

Saulnier, P 1032

Savage, PM 810

Savulescu, D 1180

Sawada, T 898

Sawyer, E 414

Scagliotti, GV 1311

Scardino, PT 678

Scélo, G 1190

Schäfer, R 361

Schafmayer, C 447, 455

Schellens, JHM 673

Schlegel, R 1549

Schmid, KW 376

Schmid, P 810

Schmidt, B 188

Schmidt, CA 447, 455

Schmidt, LS 1670

Schmiegelow, K 1670

Schöffski, P 1309

Schreiber, S 447, 455

Schrijvers, ML 1778

Schulz, C 506

Schumacher, U 602

Schuster, T 500

Schuuring, E 1778

Schüz, J 1670

Schwaiger, M 35

Schwartz, K 1676

Schwartz, PE 134

Schwertheim, S 376

Sciot, R 1032, 1769

Scolastico, C 1707

Scorilas, A 1384

Scott, R 447, 455

Scott, V 462

Sebag-Montefiore, D 1123

Sebastiani, P 1284

Seckl, MJ 810

Seebacher, V 952

Seeger, JM 1717

Seguin, S 1024

Sehested, A 1670

Seki, N 883

Selaru, P 1311

Selwood, D 541

Seneci, P 1707

Senesse, P 966

Serajuddaula, S 1657

Serio, M 685

Serot, F 966

Serra, L 1300

Serrano, S 1137

Seth, A 570

Sham, P 447, 455

Shapiro, GI 342

Shehata, M 704, 995

Shen, M-C 1185

Shepherd, CJ 1769

Shershah, S 1657

Sheu, S-Y 376

Shi, J 1618

Shimada, M 908

Shimizu, K 844

Shimizu, T 844

Shimokawa, T 325

Shiner, AM 1201

Shinto, O 844, 898

Shiozaki, A 1174, 1378

Shipley, J 1769

Shoji, K 1061

Short, D 810

Shreeves, G 1355

Shu, X 1397

Shulkin, B 1319

Side, L 1794

Sieber, O 447, 455

Silasi, D-A 134

Simes, RJ 1341

Simon, M 966

Simpson, AJG 243

Singer, G 151

Singh, N 1044

Sinicrope, FA 165

Sipos, F 765

Sirab, N 1037

Siren, MJ 833

Siren, PMA 833

Sjölin, E 1541

Sjursen, W 482

Skaar, TC 294

Skipworth, RJE 665

Skomedal, H 957

Slack, R 1428

Slagter-Menkema, L 1778

Slama, J 1284

Sleijfer, S 1699

Sludden, J 1003

Small, D 1113

Smalley, KSM 1724

Smit, EF 268

Smith, BR 1553

Smith, G 693

Smith, P 115

Smith, RA 577

Smith, SA 220

Smyrk, TC 165

Sneller, V 506

Snijders, PJF 957, 1657

Socinski, MA 1311

Solassol, J 181

Solymosi, N 765

Sondak, VK 1724

Sone, K 1061

Song, G-A 710

Sonvilla, G 1145

Soomro, IN 704, 1600

Souglakos, J 1762

Sourjina, T 475

Sova, H 1018

Spector, L 227

Spiegl-Kreinecker, S 1145

Spisák, S 765

Spraggs, CF 1371

Sprung, CN 1511

Squires, J 495

Srivastava, S 196

St George-Smith, S 577

Staffolani, S 1224

Stampfer, MJ 1079

Stark, N 1361

Statham, AL 87

Stathopoulos, E 1762

Staton, CA 594

Stättner, S 1145

Staudt, M 1213

Stearns, V 294

Steeghs, N 1699

Steele, RJC 693

Stein, K 234

Stengel, C 316

Stenman, U-H 469

Stern, PL 1129

Stewart, MH 783

Steyerberg, EW 972

Stingl, L 1578

Stockler, MR 475, 1341

Stokland, T 1670

Stone, M 1284

Storniolo, AM 294

Stovall, M 220

Strafella, E 1224

Stratford, IJ 1555

Stratford, MRL 1355

Strausberg, RL 243

Strauss, SJ 1106

Strickland, A 475

Stühmer, T 1578

Suárez, A 922

Sumitomo, M 1592

Sundquist, J 42, 1397, 1786

Sundquist, K 1397

Surowiak, P 361

Sutherland, RL 87

Sutherland, S 995

Sverdlov, ED 1533

Swanton, C 1793

Szarewski, A 1405

Szegezdi, E 754

Szekeres, T 1361

Taft, DH 419

Takai, T 916

Takeda, Y 1483

Takeshita, H 1174, 1378

Taketani, Y 1061

Talekar, G 796

Tamaro, S 1190

Tammela, TLJ 469

Tan, A 1219

Tan, BHL 665

Tan, XV 1551

Tanahashi, T 908

Tanaka, H 738

Tanaka, M 738

Tanaka, T 873

Tanemura, M 1483

Tang, K-H 332

Tang, W 570

Tanikawa, M 1061

Tanji, K 873

Tarpin, C 1081

Tascilar, M 1699

Tatagiba, M 1213

Tatarano, S 883

Tate, AR 947

Taubert, H 731

Tavassoli, FA 134

Taylor, BJ 1636

Tazzyman, S 594

Tchernitsa, O 361

Tebbutt, NC 475

Tegze, B 361

Teljeur, C 48

Tempfer, C 952

Tendo, M 898

Tenesa, A 447, 455

Tepel, J 447, 455

Terracciano, L 151

Terrier, P 1032

Terrin, L 1300

Terry, G 1405

Teschendorff, A 1294

Th Scholten, E 27

Theriault, RL 651

Theurillat, J-P 1736

Thomas, J 285

Thompson, AM 719

Thompson Coon, JS 234

Thornhill, A 1235

Thorpe, H 1099

Thorpe, S 1608

Thway, K 1769

Tian, X 188

Tian, Y 1753

Tickner, M 541

Tiling, R 35

Timmer, A 173

Tisdale, MJ 833

Toh, HC 981

Tojo, A 206

Tolle, F 1024

Tomasetti, M 1224

Tomimaru, Y 1483

Tomita, D 301

Tomlinson, IPM 447, 455

Tonita, JM 1190

Tortora, G 513

Tortoreto, M 685

Tosetto, M 456

Tóth, K 765

Toumpanakis, C 1106

Tozer, GM 1555

Tracey, E 1190

Träger, C 1670

Tranø, G 482

Trapman, J 1491

Trarbach, T 506

Traversier, S 966

Treanor, D 1519

Trentham-Dietz, A 799

Tretli, S 1786

Tripathi, A 1284

Tryfonopoulos, D 1157

Tryggvadottir, L 1786

Tsuji, S 206

Tsujiura, M 1174, 1378

Tsuruga, T 1061

Tulassay, Z 765

Turner, NC 1106

Tuupanen, S 447, 455

Tye, L 1311

Tymrakiewicz, M 414

Tzardi, M 1762

Uchida, Y 883

Ueno, NT 651

Ullrich, S 602

Umeshita, K 1483

Umezawa, K 206

Untch, M 35

Valcz, G 765

Valery, PC 620

van Dam, L 972

van Dam, P 276

Van Damme, N 837

van de Biessen, D 1699

Van de Laar, FA 48

van den Bergh, KAM 27

van den Eertwegh, AJM 803

Van der Auwera, I 276

van der Laan, BFAM 1778

van der Schouw, YT 1400

van der Straaten, T 1791

van der Veldt, AAM 803

van der Wal, JE 1778

van Doorn, L-J 262

Van Hazel, G 475

van Hinsbergh, VWM 268

van Houdt, WJ 1254

van Kemenade, FJ 1657

van Klaveren, RJ 27

Van Laere, SJ 276

van Leenders, GJLH 1491

van Leerdam, ME 972

van Noord, PAH 1400

Van Tongeren, M 1428

van Wezel, T 447, 455

Vanhuyse, M 1207

Vansteenkiste, J 301

Varga, Z 1736

Varticovski, L 1636

Vasireddy, RS 1511

Vasson, MP 966

Vatten, LJ 482

Vayshlya, NA 1533

Vedhara, K 1335

Velez, A 447, 455

Venissac, N 1627

Venkateswarlu, S 1361

Verhagen, PCMS 1491

Verheul, HMW 268

Vermeulen, PB 276

Verrill, M 1003

Verweij, J 1699

Viens, P 1081

Villanueva, C 827

Villanueva, CM 447, 455

Vincent, TJ 615

Vinogradova, TV 1533

Viola, K 1361

Vittot, M 966

Viudez, A 987

Vizoso, FJ 922

Vo, NT-P 1361

Vodicka, P 447, 455

Völzke, H 447, 455

Von Behren, J 227

von Doblen, U 796

Vonach, C 1361

Vroling, L 268

Wabinga, H 369

Wabwire-Mangen, F 262

Wacholder, S 1676

Wada, H 1483

Wada-Hiraike, O 1061

Walczak, H 1707

Walsh, CP 1080

Walsh, N 1157

Walters, SJ 1461

Walzer, S 80

Wan, S 1123

Wang, B-S 1185

Wang, D 1355

Wang, E 1311

Wang, EL 908

Wang, H 144, 862

Wang, JM 1052

Wang, L-Z 342

Wang, R 610

Wang, T 1618

Wang, X 332

Wang, Y 862

Ward, L 620

Ward, T 97, 577, 1524

Wardley, A 995

Warner, P 1503

Wasmuth, HH 482

Watanabe, M 520

Watkins, DJ 255

Watson, E 1085

Watson, S 1555

Weber, C 35

Wedge, SR 1555

Weiderpass, E 262, 1190

Welch, K 234

Welford, A 594

Weller, RE 1200

Welsh, K 97

Wen, S 144

West, CML 23, 428

West, NP 1519

Wheatley, D 1099

Whitaker, NJ 788, 1551

White, B 73

White, NMA 1244

Wichmann, B 765

Widmer, N 1198

Wijnen, JT 447, 455

Wilkinson, R 414

Willers, H 1284

Willett, WC 489, 1079

Williams, ARW 930, 1405

Williams, DC 1474

Williams, R 748

Wilson, P 249

Windschitl, H 165

Winslow, M 1461

Winter, MC 1099

Winters, U 1129

Wolf, CR 693

Wolf, H 693

Wolff, RA 144

Wöll, E 500

Wolpin, BM 1079, 1422

Wolter, P 1309

Wong, H 1391

Wong, Y-C 332

Workman, P 1555

Worm, K 376

Wrba, F 1145

Wu, K 1079

Wu, L 719

Wu, MC 1618

Wu, Y 639

Würl, P 731

Wyld, L 1461

Xiao, Y 1113

Xie, D 1618

Xie, Y 1753

Xu, C-F 1371

Xue, J 1618

Xue, Z 1371

Yaghoobi, M 237

Yamada, Y 325

Yamaguchi, K 325

Yamamoto, M 774

Yamasaki, A 738

Yang, S 1456

Yang, T-S 981

Yano, T 1061

Yao, H 1753

Yashiro, M 844, 898

Yau, T 1391

Yazdanpanah, B 1717

Ychou, M 59

Yeluri, S 1746

Yin, W 827

Yip, TT 1731

Yoon, S 710

Yoshida, K 916

Yoshimoto, K 908

Youlden, DR 620

Young, BD 1044

Young, C 1428

Yousef, GM 1244

Yu, MC 610

Yuan, J-M 610

Yuen, H-F 332

Yurtsever, H 151

Zacharakis, E 1327

Zachary, I 541

Zafar, A 1657

Zaitoun, AM 704

Zalcberg, J 475

Zanini, N 1306

Zanke, B 447, 455

Zarate, R 987

Zborovskaya, IB 1533

Zettl, A 151

Zhang, B-H 1185

Zhang, H 561

Zhang, P 862

Zhou, B 1753

Zhou, BP 639

Zhou, S 188

Ziel-van der Made, ACJ 1491

Ziller, M 645

Zinovyeva, MV 1533

Ziprin, P 1327

Zironi, S 68

Zisterer, DM 1474

Zlobec, I 151

Zucca, E 673

Zweifel, M 1355

